# Novel Clinical and Laboratorial Challenges in Aspergillosis

**DOI:** 10.3390/microorganisms10020259

**Published:** 2022-01-24

**Authors:** Raquel Sabino, Cristina Veríssimo

**Affiliations:** 1 Reference Unit for Parasitic and Fungal Infections, Department of Infectious Diseases, National Institute of Health, 1649-016 Lisbon, Portugal; cristina.verissimo@insa.min-saude.pt; 2 Instituto de Saúde Ambiental, Faculdade de Medicina, Universidade de Lisboa, 1649-028 Lisboa, Portugal

**Keywords:** *Aspergillus*, invasive aspergillosis, antifungal resistances, CAPA, IAPA

## Abstract

In recent years, research in the areas of *Aspergillus* and aspergillosis has continued to advance rapidly, including advancements in genomics, immunological studies, clinical areas, and diagnostic areas. Recently, new risk groups for the development of aspergillosis have emerged—patients with influenza- or COVID-19-ssociated pulmonary aspergillosis. The rise and spread of antifungal resistances have also become a clinical concern in some geographic areas and have drawn the attention of clinicians due to difficulties in treating these infections. In this paper, a snapshot of these issues is presented, emphasizing these novel clinical and laboratorial challenges in the aspergillosis field and focusing on their actual relevance.

## 1. Antifungal Resistances in *Aspergillus*

Global estimates have indicated that there are approximately 250,000 cases of invasive aspergillosis (IA) annually [1]. *Aspergillus* causes serious infections with a high mortality rate (more than 99% if not treated and 45% if treated) [2]. Invasive aspergillosis affects immunocompromised patients in particular and represents one of the major causes of morbidity and mortality in specific risk groups, such as patients receiving immunosuppressive therapy or chemotherapy.

**Figure 1:**
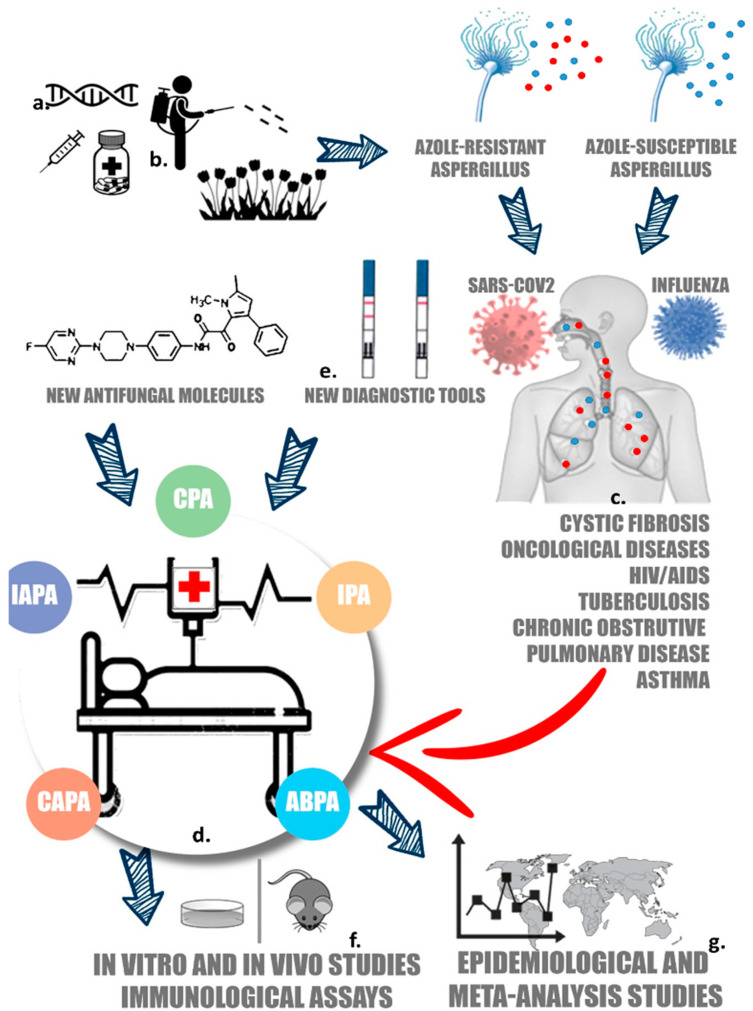
Major “hot topics” on clinical and laboratorial challenges in aspergillosis: intrinsic (**a**) and secondary (**b**) azole resistances in *Aspergillus*; (**c**) inhalation of susceptible and resistant conidia by high-risk patients; (**d**) development of *Aspergillus*-associated diseases, i.e., chronic pulmonary aspergillosis (CPA), allergic brochopulmonary aspergillosis (ABPA), invasive pulmonary aspergillosis (IPA), influenza-associated pulmonary aspergillosis (IAPA), and COVID-associated pulmonary aspergillosis (CAPA); (**e**) application of new diagnostic tools and administration of new antifungal compounds; (**f**) development of epidemiological studies; and (**g**) data from meta-analysis and the development of immunological assays.

More recently, IA has been associated with non-neutropenic patients, such as those who are hospitalized in ICUs with infections from the SARS-CoV-2 and influenza viruses. In addition, *Aspergillus* may cause chronic pulmonary aspergillosis (CPA), a progressive and generally fatal complication resulting from various lung diseases, including tuberculosis, cystic fibrosis, infections caused by other mycobacteria, and chronic obstructive pulmonary disease (Figure 1).

Patients suffering from aspergillosis are primarily treated with triazoles. Treatment with these antifungals has led to a decrease in IA mortality of 50% or more [3]. However, the emergence of azole resistance in *Aspergillus fumigatus* has raised concerns in the scientific and clinical communities [4,5,6], as it complicates the management of patients with invasive aspergillosis in the detection of resistance, treatment, and responses to the antifungal therapy, due to its association with higher mortality rates [7].

Cases of IA caused by resistant isolates have been described in all the risk patients mentioned above. The reasons suggested for increased resistance include the use of prophylactic antifungals and the use of agricultural azoles (Figure 1) [5]. The most well-known mechanisms of resistance to azoles involve mutations in the *cyp51*A gene [4]. Triazoles bind to lanosterol 14α-demethylase, a cytochrome P450 enzyme encoded by the CYP51 gene, inhibiting ergosterol biosynthesis. Azole cross-resistance has been linked to different hotspot mutations in this gene [7,8]. Other genetic alterations conferring azole resistance include tandem repeats in the promoter, associated with point mutations in the *cyp51*A gene [7,9,10]. *Aspergillus* isolates with this mutation were found in the environment and in azole-naïve patients (i.e., patients who had never been in contact with clinical azoles for treatment or prophylaxis) [11]. Exposure of *Aspergillus* (especially *Fumigati*) to azole derivatives that are commonly used as agricultural pesticides appears to have a possible high impact on azoles’ resistance. High amounts of azoles are sold annually for the purpose of plant protection, which may lead to the development of higher levels of resistance [12]. On the other hand, an emergence of non-*cyp51A-*mediated mechanisms has been observed in patients who are chronically treated with triazoles [11].

Cryptic or sibling species (within a determined *Aspergillus* section) are species that have very similar macro- and microscopic features, although they are different at the molecular level. These genetic differences may result in differences in their antifungal susceptibility and virulence patterns. Identification of these species is based on a comparative sequence of a protein-encoding locus, such as β-tubulin, calmodulin, and others [13]. Therefore, in addition to the previously mentioned acquired azole resistance in *A. fumigatus*, several cryptic or sibling species within the *Aspergillus* section *Fumigati* (e.g., *A. udagawae, A. felis,* and *A. lentulus*) [14], as well as other *Aspergillus* species in different sections, may have reduced or varied susceptibility or even intrinsic resistance to the azoles, as well as to other antifungals [15,16,17,18].

To date, most molecules developed for use in therapeutics have targeted the ergosterol in the cell plasmatic membrane (the azoles’ and polyenes’ target) and the cell wall (the echinocandins’ target). New antifungal molecules are being developed, and some have already been included in clinical trials (Figure 1). These antifungals show activity against the cell membrane metabolic pathways, the fungal cell wall, novel CYP inhibitors, and drugs targeting cell signaling pathways (Table 1) [19,20,21,22].

## 2. IAPA (Influenza-Associated Pulmonary Aspergillosis) and CAPA (COVID-Associated Pulmonary Aspergillosis)

IAPA and CAPA are newly described clinical manifestations of pulmonary aspergillosis that cause high mortality in patients, most of whom were previously immunocompetent. Viral, fungal, and host factors are involved in the pathogenesis of IAPA and CAPA. However, this process is not fully understood [23]. Both IAPA and CAPA are potentially lethal complications in critically ill patients. It is thought that the number of described cases is lower than the real number of cases, with an evident sub-valorization of the real scenario [24,25].

In 2018, Schauwvlieghe et al. [26] described for the first time the occurrence of pulmonary aspergillosis in patients diagnosed with influenza infection during seven consecutive influenza seasons. In that study, 19% of the 432 patients admitted to the ICU were diagnosed with IAPA.

By using experimental models, Seldeslachts et al. [27] confirmed that influenza is an independent risk factor for the development of IAPA in an immunocompetent host, showing that IAPA develops in immunocompetent mice even without corticosteroids.

Regarding CAPA, the hospital mortality observed in 192 patients was highly variable, with an overall mortality rate of 48.4%, ranging between 22.2% and 100% [28].

Different numbers on mortality associated with CAPA have been published. This discrepancy can most likely be explained by differences in antifungal treatment strategies and by the limited number of patients with CAPA who were enrolled in those studies [29]. The incidence of CAPA varies with differences in diagnostic criteria, methods, definitions, and local practices. According to a meta-analysis performed by Mitaka et al. [29], approximately 10% of mechanically ventilated patients with COVID-19 who were hospitalized in an ICU were affected by CAPA [28].

IAPA and CAPA share some similarities, such as the prevalence of IPA (invasive pulmonary aspergillosis) in COVID-19 cases as well as in cases of influenza-associated acute respiratory distress syndrome, similar clinical courses in ICU (with a trend of a longer median interval between ICU admission and CAPA diagnosis), and the background of patients. The major differences between CAPA and IAPA are the following: (i) a higher proportion of older patients among CAPA patients; (ii) a lower proportion of patients with radiological findings suggestive of IPA among CAPA patients; (iii) a lower proportion of ECMO among CAPA patients; and (iv) an earlier onset of IAPA after ICU admission (IAPA develops at a median of 3 days after ICU admission, whereas CAPA develops after a median of 4–8 days after ICU admission or intubation) [23,24].

Severe influenza promotes the destruction of the respiratory epithelium, compromising the ciliary function necessary to eliminate *Aspergillus* conidia and leading to extensive damage to the pulmonary epithelium, the mechanical barrier against invasive aspergillosis. Common features associated with IAPA include epithelial damage, NADPH-oxidase deregulation, and the modulation of immune function caused by the virus [30]. The receptor SIGLEC-15 (Sialic Acid Binding Ig Like Lectin 15) is expressed on the surface of immune cells. This receptor is usually bound and linked to sialic acids that are present in mammalian cells. In the presence of neuraminidase, this receptor binds to non-self sialylated ligands. According to Dewi e Cunha [31], the receptor SIGLEC-15 may bind to sialic acids that exist on the cell wall surface of *A. fumigatus* conidia. Therefore, neuraminidase inhibitors, such as oseltamivir, might impair antifungal responses and predispose individuals with severe influenza to invasive aspergillosis.

Another view was proposed by Seldeslachts et al. [27], who demonstrated that early oseltamivir treatment regulates the severity of influenza and diminishes susceptibility to IAPA in a time-dependent manner. Influenza induces a strong IFN-γ/Th1 response by lessening the macrophage phagocytic activity in an influenza A *fumigatus* model. In an early stage of influenza infection, oseltamivir treatment reduces the viral burden, allowing the restoration of the phagocytic function, decreasing the severity of the infectious pathology, and facilitating an active antifungal clearing process.

SARS-CoV-2 enters the respiratory epithelium via the SARS-CoV-2 *ACE2* receptor but causes lower epithelial damage than influenza. It was observed that SARS-CoV-2 causes diffuse alveolar and endothelial vascular cell damage associated with an increase in the capillary permeability and fluid leakage, leading to congestion, edema, and diffuse inflammatory infiltrates, and even causing pulmonary tissue necrosis [32]. Given the possible association between the use of corticosteroids (such as dexamethasone) and CAPA, it is advisable to intensify the screening for invasive aspergillosis in this group of patients. The use of immunomodulatory drugs, hospitalization in wards with no appropriate room ventilation or air changes, and previous lesions in patients’ lungs may also predispose patients to CAPA [33].

As IAPA and CAPA cases exhibit atypical clinical features (i.e., typical host factors and radiological features for invasive aspergillosis may not exist), Feys et al. [25] recommended that, after ICU admission, all influenza patients who are mechanically ventilated should be considered for a bronchoscopy to visually inspect the airways and to sample bronchoalveolar lavage (BAL) for fungal culture and galactomannan (GM) testing [32].

In CAPA patients, serum biomarkers are often negative, making the distinction between colonization and angio-invasive disease difficult. Detection of GM in serum presents 82% sensitivity and 81% specificity in neutropenic critically ill patients with proven IPA [28]. On the other hand, the detection of GM in BAL has an equivalent or greater sensitivity compared to its detection in serum [34], with GM detection in BAL being more indicated in non-neutropenic patients, such as COVID-19 patients [35,36]. According to a meta-analysis presented by Chong et al. [28], BAL GM is 88–90% sensitive in both neutropenic and non-neutropenic groups of patients. The lack of positive cases of GM in serum encountered in non-neutropenic COVID-19 patients can be explained by multiple potential etiologies [28]. Due to the cost, response times, and the technical equipment necessary to perform GM detection, several diagnostic tests for IA have been developed, including the so-called “point of care” tests that were developed as immunochromatography tests (Figure 1), such as the *Aspergillus* GM lateral flow assay (GM-LFA) [37].

More recently, other AI-associated antigens have been used to produce more rapid diagnostic tests. The *Aspergillus*-specific lateral flow device (LFD) is an immunochromatographic assay that detects an extracellular glycoprotein antigen (tetra (1→5)-β-d-galactofuranoside) circulating in BAL and secreted during the active growth of the fungus [38]. Given the need for very fast answers for IAPA and CAPA patients, these tests represent an advance in the diagnosis of aspergillosis, as they can sometimes be performed at the bedsides of patients.

## 3. Concluding Remarks

Given this small snapshot of recent publications, what are the major concerns that need to be addressed? Figure 1 summarizes the major “hot topics” on clinical and laboratorial challenges in aspergillosis and the issues that are under development and active research, in addressing that question.

The proportion of influenza-infected and COVID-19 patients who are co-infected, super-infected, or colonized with *Aspergillus* is unclear Due to this lack of awareness, we may have underestimated the occurrences of viral infection-associated IA diseases, because IA would not likely be suspected. Therefore, many more meta-analysis studies should be performed to gain an understanding of the real scenario that is involved. Due to the lack of awareness, relevant laboratory tests for IA are not likely to be ordered, and clinical diagnosis and treatment for IA is likely to be delayed or not carried out.

Azole resistance is complex and influenced by a multiplicity of factors. To date, resistance mechanisms have not been fully characterized in *A. fumigatus*, and there is much to explore in other *Aspergillus* species. Given the worldwide rise of azole resistance in this genus, a central registration of the treatment and outcome data for patients with resistant *Aspergillus* disease is needed. Studies of the virulence attributes that enable the success of the infection treatment process are needed as well, to better understand the mechanisms beyond the infection process in different patients.

## Figures and Tables

**Table 1:** New antifungals under development or on clinical trials and their classes, activities and cellular targets.

	Antifungal Drugs in Clinical Development	Characterization	Activity	Target
**Agents targeting** **cell wall integrity**	Rezafungin	Echinocandin	*Candida* spp., including resistant isolates	(1–3) β-D-glucan synthase
	Ibrexafungerp	Triterpinoid	Broad spectrum—*Candida* spp., including resistant isolates and moulds	
	Fosmanogepix	Gwt1 Inhibitor	Broad spectrum—*Candida* spp., including resistant isolates and moulds with reduced susceptibility to current antifungals	Glycosylphosphatidyl inositol
	Nikkomycin Z	Nikkomycin	Dimorphic fungi	Chitin synthase
**Agents targeting cell membrane**	VT-1129	Tetrazole	*Cryptococcus* sp.	Lanosterol 14α demethylase (CYP51)
	Oteseconazole	Tetrazole	*Candida* spp.	
	VT-1598	Tetrazole	*Coccidioides*, other dimorphic fungi and moulds	
	SUBA-ITC	Triazole	Same as Itraconazole	
	Encochleated Amphotericin B (MAT2203)	Polyene	Fungal infections in the central nervous system	Fusion with cell membrane
	Aureobasidin A	Cyclic depsipeptide antibioti	* Candida * spp., other species of yeasts, some *Aspergillus* spp.	Inositol phosphorylceramide synthase
**Agents with** **intracellular targets**	Olorofim	Orotomide	Broad-spectrum activity against filamentous and dimorphic fungi	Dihydro-orotate dehydrogenase
	VL-2397	Cyclic hexapeptide siderophore	*Aspergillus* (except *A. niger*), *C. glabrata* and *C. kefyr*, *Cryptococcus*, and *Trichosporon asahii*	Iron metabolism siderophore ferrichrome.
	T-2307	Arylamidine	*Candida* spp., *C. neoformans, C. gattii,* *Malassezia furfur*, and *Fusarium solani*	Mitochondrial membrane
	MGCD290		*Candida* spp., *Aspergillus* spp., *Mucor,* and *Fusarium*	Histone deacetylase
	***Drugs used for other purposes,*** ***now tested for antifungal activity***			
	AR-12	Antitumor celecoxib-derivative	Broad spectrum activity	Acetyl-CoA inhibition
	Tamoxifen	Selective estrogen receptor modulators	*Cryptococcus*	Calmodulin inhibition
	Sertraline	Antidepressant— group selective serotonin reuptake inhibitors	*Cryptococcus*	Protein synthesis via translation initiation factor
	Aurofin	Antirheumatic agent	*C. albican*s. Biofilms, some moulds	Oxidative cell death
